# Origin and extension of the IFT complex in early eukaryotic evolution

**DOI:** 10.1186/2046-2530-1-S1-P56

**Published:** 2012-11-16

**Authors:** J van Dam, MC Field, M Huynen

**Affiliations:** 1 CMBI, NCMLS, Radboud University Medical Centre, the Netherlands; 2 Department of Pathology, University of Cambridge, UK

## Background

The intraflagellar transport (IFT) complex is an ancient protein complex that facilitates active trafficking of proteins and molecules across the eukaryotic cilium. Based on similar domain compositions Avidor-Reiss et al. (2004) and Jékely et al. (2006) postulated that the IFT originates from an ancestral proto-coatomer complex that also gave rise to vesicle coating complexes (e.g. COPI, COPII, Clathrin) and the Nuclear Pore Complex. Using comparative genomics we provide direct phylogenetic evidence of the proto-coatomer origin of the IFT.

## Results

We identified the COPIα, -β2 and –ε subunits as closest paralogs to 12 IFT subunits comprising all three sub-complexes (IFT-A, -B and the BBSome). Our analysis suggests that IFT-A and the BBSome arose from an IFT-B like proto-IFT complex by intra-complex duplication of subunits. We show that the BBSome is a modular component that is lost in eukaryotic species as a precursor to ciliary loss in organisms such as fungi, apicomplexa and plants.

## Conclusions

Identification of the proto-coatomer origin and subsequent evolution of the IFT complex strengthens the suspected involvement of IFT components in vesicle transport and provides a rationale for its mechanism. Expansion of ancestral subunits by duplication as well as co-evolution of specific subunits provides some insight on modularity and internal structure of the IFT complex.

